# The Voltage-Dependent Anion Selective Channel 1 (VDAC1) Topography in the Mitochondrial Outer Membrane as Detected in Intact Cell

**DOI:** 10.1371/journal.pone.0081522

**Published:** 2013-12-06

**Authors:** Marianna F. Tomasello, Francesca Guarino, Simona Reina, Angela Messina, Vito De Pinto

**Affiliations:** 1 Department of Biological, Geological and Environmental Sciences, Section of Molecular Biology, University of Catania, and National Institute for Biomembranes and Biosystems, Section of Catania, Catania, Italy; Hertie Institute for Clinical Brain Research and German Center for Neurodegenerative Diseases, Germany

## Abstract

Voltage-Dependent Anion selective Channel maintains the permeability of the outer mitochondrial membrane and is relevant in bioenergetic metabolism and apoptosis. The structure of the protein was shown to be a β-barrel formed by 19 strands. The topology or sideness of the pore has been predicted with various approaches but a general consensus was never reached. This is an important issue since VDAC is considered receptor of Hexokinase and Bcl-2. We fused at VDAC1 C-terminus two tags separated by a caspase cleavage site. Activation *in cellulo* of caspases was used to eventually separate the two reporters. This experiment did not require the isolation of mitochondria and limited the possibility of outer membrane rupture due to similar procedures. Our results show that the C-terminus end of VDAC faces the mitochondrial inter-membrane space.

## Introduction

The voltage-dependent anion channel (VDAC) is the most abundant integral membrane protein of the mitochondrial outer membrane (MOM) where it forms hydrophilic pores [1,2]. It is considered a key regulator of metabolite flows as the preferred exchange route of adenosine nucleotides from and to the mitochondrion [3]. VDAC is also co-responsible in various cell processes including apoptosis [4], calcium homeostasis [5] and diseases such as cancer [6]. Deficiency of VDAC has been associated with a lethal encephalomyopathy [7]. The structure of VDAC has been disclosed in 2008, when three independent groups reported to be an antiparallel β-barrel containing 19 amphipathic β-strands joined to an N-terminal sequence, containing amphipathic alpha-helix segments [8-10]. The three works showed noticeably differences in the N-terminal assignment, also because the first of them was determined by NMR at room temperature [8], the second with a mixed crystallographic and NMR strategy [9] and the third one by pure crystallography [10]. A report questioning this structure and proposing a different arrangement of the protein [11], was confuted by the three joined groups, reaffirming the 19-barrel structure [12]. In the following text, for the sake of simplicity, we will refer to the crystallographic structure proposed in [10], but it is important to remember that also the model by Colombini [11] place the C-terminus alike in the membrane.

The VDAC transmembrane β-barrel is characterized by short turns from one side of membrane and longer loops from the other [8-10]. The sideness of connecting segments has not been definitely assigned, since various and contradictory results were reported [13-15]. This is a very important issue, since VDAC is considered to be the dock for various proteins, notably Hexokinase (HK) [16] and Bcl-2 [17]. Therefore, the knowledge of the correct topography is a prerequisite to any experimental or predictive docking assay for VDAC.

**Figure 1:**
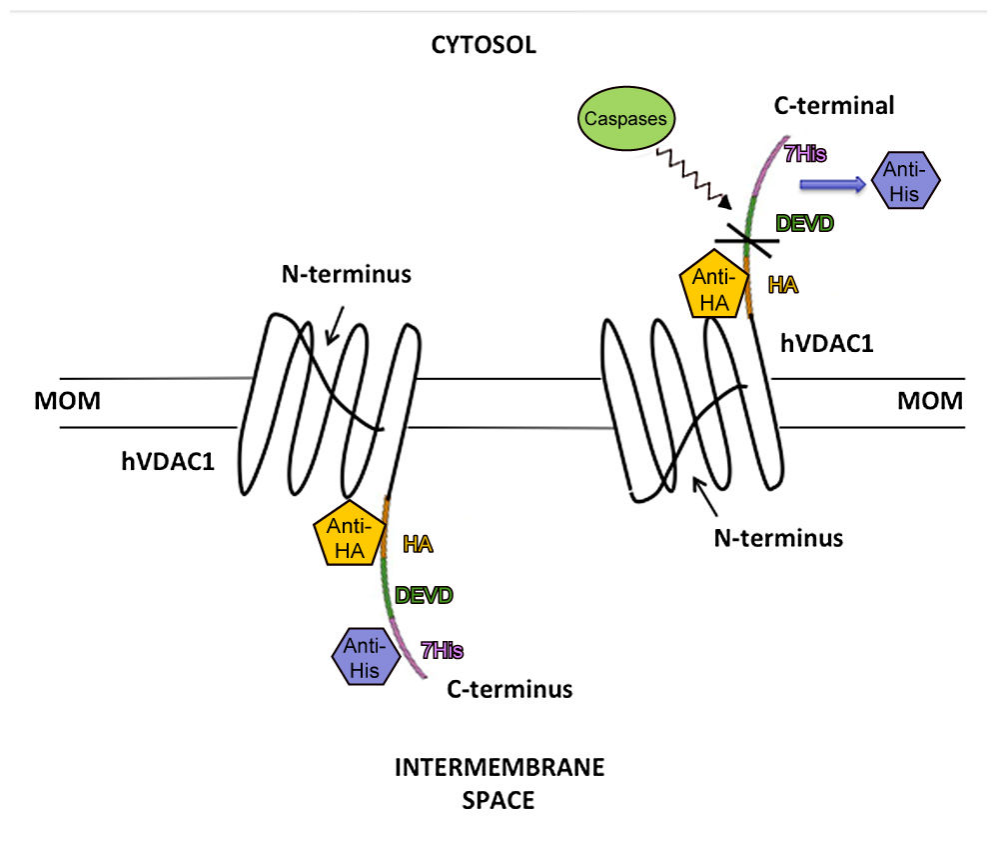
Experimental strategy adopted in this work to prove the topography of VDAC. In the figure the possible output of caspase's cleavage is shown. Right side: the C-terminus faces the cytosol, exposing the DEVD to caspase's cleavage and causing the removal of the His tag. Left side: the opposite topography will not cause any separation of the two tags.

To date, only biochemical strategies have been used to validate the accessibility of specific sequences in VDAC: exposed domains were looked for with antibodies and proteases [13-15]. These experiments and, in particular, anti-peptide Abs, were designed using predictive bioinformatic models, since the three-dimensional structure was not available. At the same time the exposure of connecting turns/loops was assayed on isolated mitochondria, with the aim to assign the accessible epitope to the external or internal side of the outer membrane. The main technical problem was the difficulty to preserve and prove the entirety of the outer membrane in isolated mitochondria. In the present study, we describe a new approach to solve the question of VDAC’s topography using confocal microscopy. This allowed us to getting information in single intact cells. Briefly, our strategy is based on the generation of a recombinant probe capable to convert subcellular spatial signals into steady “all-or-none” fluorescence intensity responses [18]. Since the C-terminal transmembrane β-strand has amino acids just sufficient to span the membrane, we have attached at this extremity the following boxes: a Ha tag, then the peptide DEVD (consensus site for caspases 3/7) and a 7xHis tag. This probe is expected to protrude outside the membrane and to be accessible. The activation of caspases in the cytosol upon staurosporine exposure is predicted to cleave at the DEVD sequence, if accessible from outside, thus releasing the 7xHis tag and separating the fluorescence signals produced from the two reporters. Figure 1 shows the rationale of this strategy.

## Experimental

### Cell culture and transfection

HeLa cells were cultured in 5% CO_2_ in DMEM (Invitrogen) supplemented with 10% FBS and 1% penicillin/streptomycin (Invitrogen). Once the cells reached 95% confluence, they were split (using 0.25% trypsin-EDTA; Invitrogen) into fractions and propagated or used in experiments. Cells were passaged biweekly. Passages 10–50 were used in our experiments. For plasmid transfection, cells were plated directly on 6 wells plate or (according to the considered experiment) on 13 mm diameter round glass coverslips inside 12 well plates. When reaching 80% of confluences, cells were transiently transfected by using Lipofectamine 2000 (Invitrogen), as described in the manufacturer’s instructions.

### Plasmidic constructs

The recombinant construct hVDAC1-HaDEVDHis carrying a C-terminal tag including, in sequence, the hemagglutinin tag, the specific caspase 3 cleavage site and 7 histidine residues (Ha-DEVD-7His) was obtained cloning MluI/NotI a 48 bp DNA fragment coding the sequence DEVD-7His into a plasmid expressing hVDAC1-Ha. DEVD-7His fragment was obtained annealing the forward and reverse C-term-H1-HA oligonucleotides (C-term-H1-Ha fw: 5’-CGC GTG TAG ATG AAG TCG ATC ACC ACC ACC ACC ACC ATC ACT AAG C-3’, C-term-H1-Ha rev: 5’-GGC CGC TTA GTG ATG GTG GTG GTG GTG GTG ATC GAC TTC ATC TAC A-3'); conditions: 95°C 5 min, 92°C 2 min, ramp cool to 25°C, 4°C. The coding sequence was inserted downstream of the CMV promoter into the mammalian expression vector pcDNA3 and pCMS-EGFP (Clontech). A modified version of the same vector, pCMS-mtRed, was also used to clone HVDAC1HaDEVDHis. pCMSmtRED expresses the Red fluorescent protein from the coral *Discosoma* *sp*. The mt-targeting sequence is the Cytochrome Oxidase subunit III targeting sequence that addresses the fluorescent protein to the mitochondrial matrix. The GFP-DEVD-RR probe was generated by insertion of the DEVD coding sequence by site-directed mutagenesis on pcDNA3.0-GFP-RR (also named here MomGFP) (kindly provided by Borgese, [19]) by the Quick Change Site-directed mutagenesis kit (Stratagene) using primers DEVD fw and DEVD rev (DEVD fw 5’-GGT GGA GGA GGT TCA GAT GAA GTC GAT TCA GGA GGA GGT GGA TC-3’; DEVD rev 5’-GAT CCA CCT CCT CCT GAA TCG ACT TCA TCT GAA CCT CCT CCA CC-3’). This modified MomGFP was called MomDEVDGFP in this work. 

### DEVD cleavage

Cells transfected with pCMSmtRedVDACHaDEVDHis or MomDEVDGFP or both were exposed to 0.1-1 mM of staurosporine to induce caspases activation. For each determination, the time needed to efficiently reach the cleavage of the DEVD was established by following the behaviour of the MomDEVDGFP, since the DEVD cleavage caused the GFP to be redistributed in the cytosol. When GFP became diffused in 90% of the population in the coverslips, cells were fixed and immunostained to reveal the HA and His tags. 

### Selective permeabilization of plasma membrane and mitochondrial membranes

Permeabilization of cellular membranes was obtained by incubation of cells (adherent to the coverslip) in an intracellular buffer (130 mM KCl, 10 mM NaCl, 20 mM 4-(2-hydroxyethyl)-1-piperazine ethanesulfonic acid (HEPES), 1 mM MgSO_4_, 5 mM succinate pH 7.2) supplemented with 50 µM ethylene glycol tetra-acetic acid (EGTA) and the appropriate concentration of the detergent digitonin. EGTA was added to avoid Ca^2+^ induction of the permeability transition in the mitochondria of permeabilized cells. Permeabilization of mitochondrial membrane was achieved by using Triton X-100 instead of digitonin.

Increasing concentrations of digitonin and Triton X-100 were tested for their efficacy to selectively permeabilize plasma or mitochondrial membranes, respectively. The tested concentrations were from 10 to 60 µM digitonin and 0–0.2% (v/v) Triton X-100. Stock solutions were prepared in PBS. Selective permeabilization of plasma or mitochondrial membranes in transfected cells was carried out by adding respectively 40 µM digitonin or 0.05% (v/v) Triton X-100 in intracellular buffer for 2 min. The loss of plasma membrane permeability was followed in live by microscopy, estimating the leakage of cytosolic GFP in >95% of cells while the immunostaining of the endogenous cytochrome c was used as a control of the mitochondrial outer membrane integrity.

### Fluorescence protease protection assay

Trypsin or proteinase K could enter digitonin- or Triton-permeabilized cells and digest the cytosolic moiety of proteins anchored to the outer side of the mitochondrial outer membrane. The digestion of this cytosolic moiety of the MomGFP, resulting in the loss of the green fluorescence, was exploited to establish the time needed for the eventual digestion of the His and/or HA tags, fused to the hVDAC1 chimera. The assay was carried out as previously reported [20]. Optimal results were obtained by using 100 µg/ml proteinase K for 5 min.

### Immunostaining

Cells were fixed in 3.7% formaldehyde for 20 min at room temperature before permeabilization in 0.3% (v/v) Triton X-100. Unspecific binding was blocked by incubation in 0.2% gelatin/PBS, and staining was performed using specific primary antibodies against the HA and the His tags and against the endogenous cytochrome c (Santa Cruz). After washing, cells were incubated with appropriate secondary antibodies (anti mouse Alexa 680, anti rabbit Alexa 546 or Alexa 488 from Molecular Probes). Coverslips were mounted with Prolong Gold (Invitrogen) and examined under a FV1000 confocal microscope using the Fluoview Olympus image software (version 1.8).

### Mitochondrial membrane potential detection

To detect mitochondrial membrane potential, cells were incubated with MitoTracker Red, which passively diffuse across the plasma membrane and accumulate in functional mitochondria. Once mitochondria were labeled, the cells were fixed and immunostained, as MitoTracker Red is also retained after Triton permeabilization. Analysis was performed on an Olympus Fluoview 1000. 

### Microscopy Image Analysis

Imaging of living or fixed immunostained cells was carried out on an Olympus Fluoview FV1000 confocal microscope, using a 63 Plan-Apo/1.4-NA oil-immersion objective. Standard 3 confocal channels (3 Photomultiplier detectors) acquisitions were made by using the following lasers, mounted on a laser combiner: multi-line Argon laser (457 nm, 488 nm, 515 nm), total 30 mW, HeNe-Green laser (543 nm), 1,5 mW, HeNe-Red laser (633 nm), 10 mW. Ten random fields for each well were imaged, with each treatment repeated twice in three separate experiments. Single optical sections (1-µm *z* axis) through the middle of the cells were acquired for each field. The pinhole was adjusted to keep the same size of *z*-optical sections for all analysis carried out. Sequential mode imaging was performed to ensure that there was no crosstalk between the channels. The Pearson co-localization coefficient (*R*) was determined using the Olympus FV 1000 co-localization software (release 1.8). Fluorescence was quantified using both the FV1000 and NIH Image J single particle analysis software. Images were assembled using Adobe Photoshop and Power Point.

## Results

### The construct VDAC1HaDEVDHis is targeted to mitochondria and the C-terminus is not accessible to cytoplasmatic caspases

**Figure 2:**
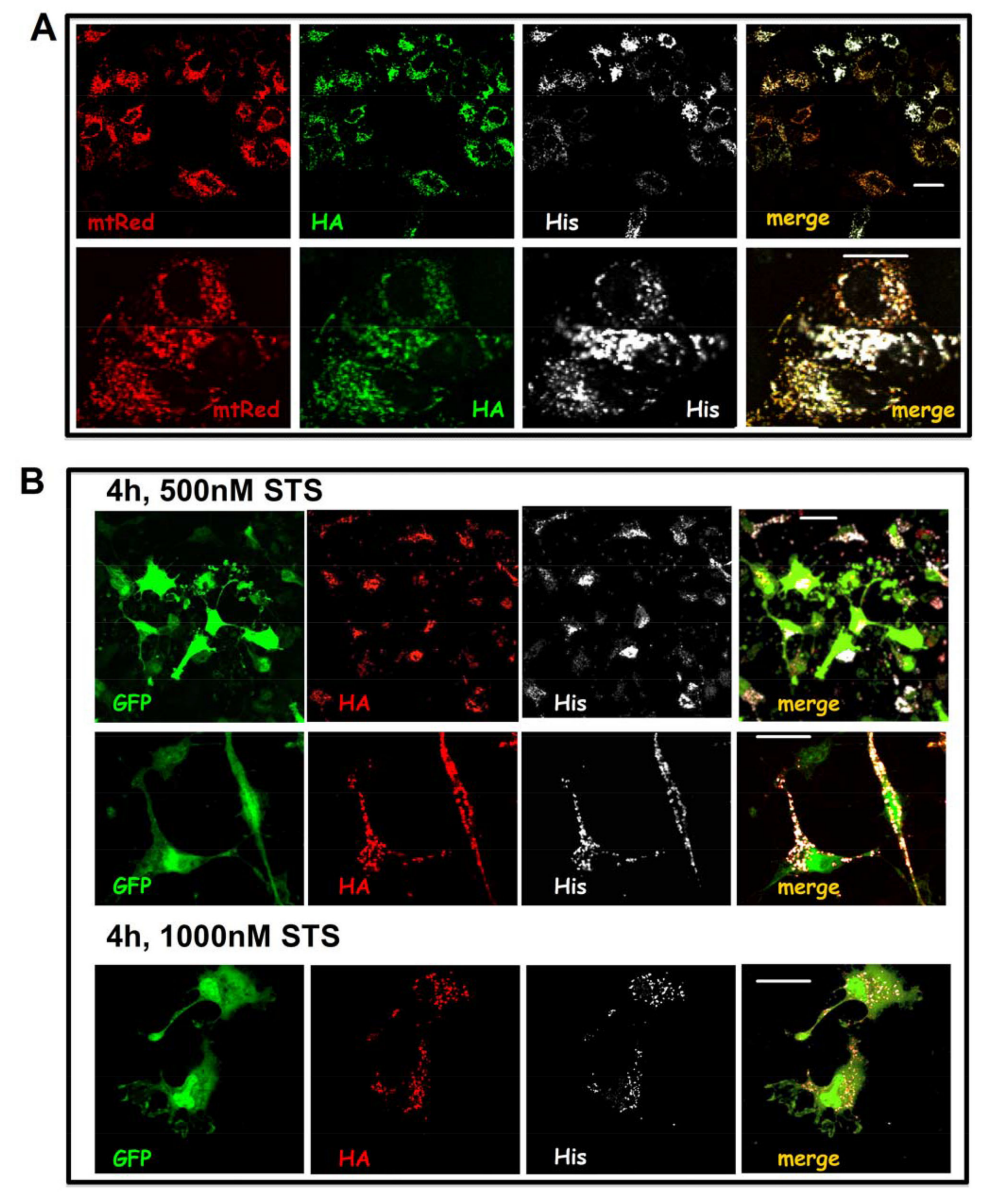
HA and His immunostaining defines the position of the *C-*terminal end of VDAC1 in the cell. **A**) **Expression and sorting of the chimera VDAC1HaDEVDHis**. HeLa cells were transfected with pCMSmtDsRed-VDAC1HaDEVDHis and double-immunostained for the HA and the His tags. pCMSmtDsRed-VDAC1HaDEVDHis transfection resulted in the simultaneous expression of both mitochondrial hVDAC1-HaDEVDHis (HA green and His white) and mitochondrial mtDsRed (red) used as transfection and targeting reporter. Fluorescence distribution analysis indicates the co-localization of both the HA and the His tags with the mtDsRed in the mitochondrion. Images are representative of 50 cells analyzed for each condition in experiments performed in triplicate. The lower panel is a magnification. Scale bar, 20 µm. **B**) **Caspase activation does not separate HA from His tag, indicating that the DEVD is not accessible**. HeLa cells transfected with pCMSGFP- VDAC1HaDEVDHis were exposed to staurosporine, fixed and double immunostained for the HA and the His tags. Cells expressing the cytosolic GFP (green), positively stain for both the HA (red) and His (white) tags, even upon mild staurosporine exposure. In the upper panel cells treated with 500 nM staurosporine are shown. In the lower panel the staurosporine was used at 1 µM. Note the dramatic morphological modification induced by staurosporine. Images are representative of 50 cells analyzed for each condition in experiments performed in triplicate. Scale bar, 20 µm.

In order to probe the orientation of VDAC we produced a chimeric cDNA, the hVDAC1-HaDEVDHis and inserted it in the double promoter plasmid pCMSmtRed-VDAC1HaDEVDHis that allows for the simultaneous expression of hVDAC1-HaDEVDHis and the mtDsRed here used as transfection and targeting reporter. The merge in Figure 2A confirms that the construct is efficiently targeted to mitochondria and that both tags are accessible to immunostaining. Co-localization of our construct with the mitochondrial reporter mtDsRed and the endogenous protein cytochrome c, present in the IMM space, is a strong indication of the correct insertion of the construct (Figure S1). We have shown in previous work [21] that in similar conditions overexpressed hVDAC1 co-localizes with mitochondrial activated Bax that is targeted to MOM.

Upon staurosporine exposure, caspases are activated in the cytosol [22]. The DEVD sequence is a consensus target site for the proteolytic action of caspase 3 and 7. Thus, if the VDAC C-terminus is exposed to the cytosolic side, we expected to lose the His tag as a result of the DEVD cleavage. Consequently only the HA should be detected at mitochondrial level. Conversely, in the opposite orientation, the DEVD will be not accessible to caspases, therefore the HA and His tags will be detected in mitochondria (Figure 1). 

**Figure 3:**
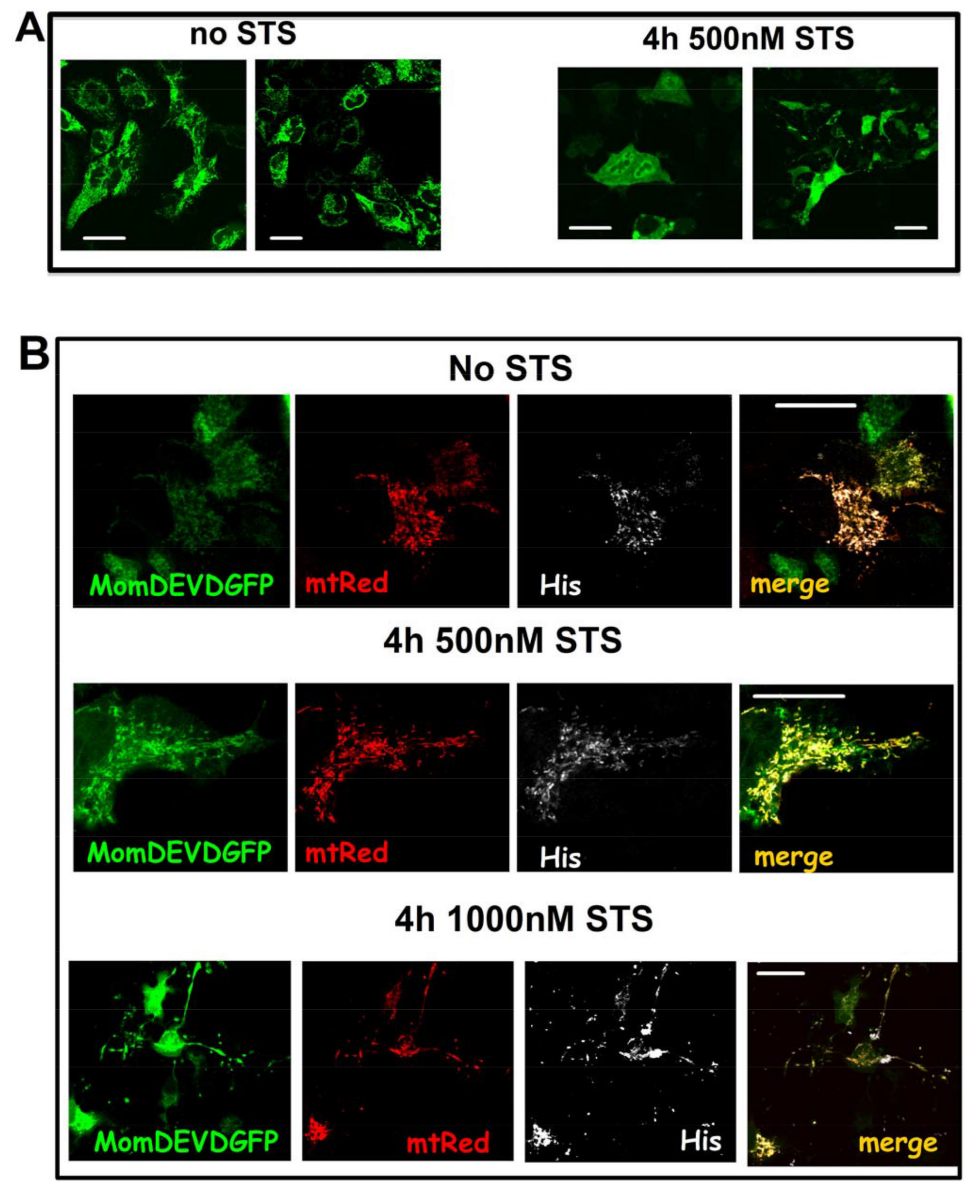
DEVD is cleaved in MomDEVDGFP upon staurosporine exposure. **A**) HeLa cells were transfected with the chimera MomDEVDGFP. In non apoptotic cells, the GFP localizes in the mitochondrial outer membrane facing the cytosol. When apoptosis was induced by treatment with 0.5 µM staurosporine, the GFP fluorescence become cytosolic, as a result of the DEVD cleavage. **B**) Co-transfection of HeLa cells with both pCMSmtDsRed-VDAC1HaDEVDHis and MomDEVDGFP. Images were obtained after fixation and immunostaining for the His tag (white). Red stains for DsRed (mitochondria), green for GFP. The upper row shows untreated cells, where all the three overexpressed proteins (mtDsRed, MomDEVDGFP and hVDAC1-HaDEVDHis) localize into the mitochondria. Growing concentrations of staurosporine (caspase activation, lower rows) results in diffusion of the GFP fluorescence form mitochondria to the cytosol. Nevertheless, in the same condition the His tag was not lost, confirming that hVDAC1-HaDEVDHis is still in mitochondria and not accessible to caspases. Images are representative of 50 cells analyzed for each condition in experiments performed in triplicate. Scale bar, 20 µm.

Figure 2B shows immunofluorescence from the His and HA tags obtained upon addition of 0.5 or 1 µM staurosporine for 4 h. We used staurosporine, a known apoptotis inducer, because of its mild and progressive action on the cell [22]. The milder staurosporine stimulation (0.5 µM) used here to activate caspases did not cause the ΔΨ collapse (Figure S2). Accordingly with results shown in [22], 0.5 µM staurosporine induced cytochrome c release without affecting ΔΨ (Supplem. Figure 2B) while ΔΨ collapse eventually took place downstream caspases activation in cells treated with 1 µM staurosporine (Figure S2C). The concentration of 0.5 µM staurosporine at the indicated time is sufficient to activate caspases, as reported (see, i.e., Figure 3E in our work [21]) but it is not able to destroy the active membrane potential as shown by the red staining of mitotracker Red (Figure S2B). 

In the experiment shown in Figure 2B, the His and HA staining are not affected and overlap. This is a clear indication that the C-terminus is oriented towards the IMS. It is important to know that also the HA tag is a potential cleavage site for caspases, since it contains the sequence DXXD, previously found as a caspase-sensitive site [23]. This observation strengthens the sensitivity of the VDAC1 added tag HaDEVDHis to caspases.

The accessibility of the DEVD sequence to caspases was assayed with an additional control construct: the recombinant MomDEVDGFP, a chimera where the GFP is fused through the synthetic DEVD cleavage site to a MOM anchoring domain [19]. This construct is a caspase’s biosensor [18]. In living HeLa cells expressing MomDEVDGFP, the exposure to staurosporine caused the diffusion of the GFP to the cytosol (Figure 3A). Living HeLa cells expressing both MomDEVDGFP and hVDAC1-HaDEVDHis were monitored for the release of the GFP signal from mitochondria to the cytosol. When GFP became diffused in 90% of the population in the coverslips, cells were fixed and immunostained revealing that the HA and His tags were still in place, inside the mitochondria (Figure 3B shows the case for the His immunostaining). This strongly suggests that the DEVD in VDAC1-HaDEVDHis is not accessible because it is not exposed to the cytosol; in fact even though the GFP is a quite large protein, this does not prevent the cleavage of the DEVD when it is exposed to the cytosolic compartment as in MomDEVDGFP.

### FPP assay confirms that the VDAC1 C-terminus is not accessible from cytoplasm

The assay termed FPP (fluorescence protease protection) [20] has been described as a tool for determining the topological distribution of a given protein in living cells. The assay provides fluorescence readout, in response to the protease-induced loss of tags attached to a protein of interest before and after plasma membrane permeabilization. Using this method one can determine whether a protein is membrane-associated, cytoplasmic or luminal and which part of a membrane protein faces the lumen and the cytoplasm. Here we have exploited the FPP assay in order to establish the topography of VDAC. 

Digitonin interacts with cholesterol molecules causing the plasma membrane to become perforated [24] so that exogenous molecules, like proteases, can diffuse into the cell. Membranes of intracellular organelles have much lower concentrations of cholesterol than plasma membranes and are unaffected when appropriate digitonin concentrations are used [25]. Moreover EGTA is added to the intracellular buffer to avoid Ca^2+^-dependent induction of the permeability transition in the mitochondria of permeabilized cells.

When entering digitonin-permeabilized cells, proteinase K or other proteases like trypsin are able to digest cytosolic protein or the cyto-exposed moiety of proteins anchored to the mitochondrial outer membrane. Cells expressing the chimera hVDAC1-HaDEVDHis were exposed to proteinase K or trypsin, before and after plasma membrane permeabilization. In this occurrence, the tag moiety protruding into the cytoplasm is subject to proteinase K or trypsin, thus such tag will be lost. On the contrary, if the tag faces the mitochondrial intermembrane space it will not be available to the protease, then it will be detectable by immunofluorescence.

**Figure 4:**
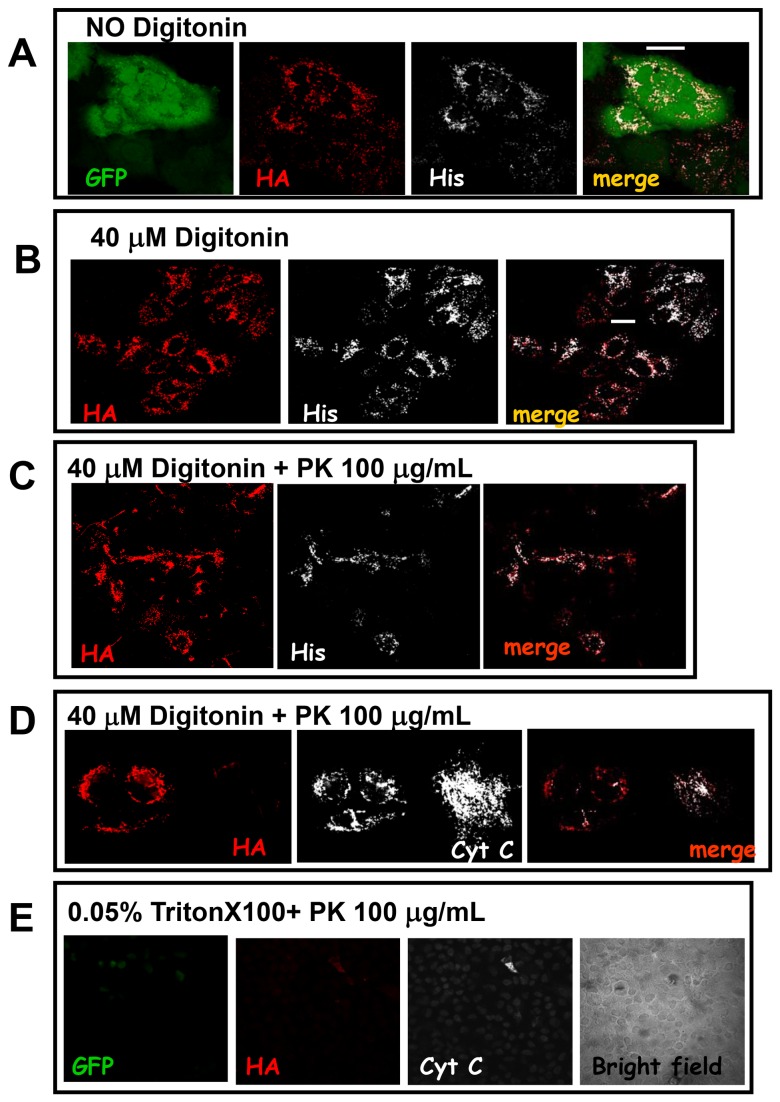
HA and His tags of chimeric hVDAC1-HaDEVDHis are protected from proteases in the FPP assay. **A**) HeLa cells transfected with pCMSGFP-VDAC1HaDEVDHis were double immunostained for the HA tag (red) and the His tag (white). **B**) Addition of 40 µM digitonin caused the diffusion of the GFP from the cytosol to the external medium. **C**) Addition of 40 µM digitonin plus proteinase K does not affect the HA and His staining. **D**) The same experiment, but cytochrome c (white) was stained instead of His tag. The stable localization of cytochrome c demonstrates that, in the condition here used, the intermembrane space is not accessible to proteases. The reduced cytochrome c signal in hVDAC1-HaDEVDHis transfected cells is just random. Images are representative of 50 cells analysed for each condition in experiments performed in triplicate. Scale bar, 20 µm. **E**) Addition of Triton X-100 damages the outer membrane and results in the entrance of proteinase K in the mitochondrial intermembrane space and in the digestion of HA and His tags. The picture represents the HA and cytochrome c staining. The Bright field is the same cell field, showing the presence of cell, even though they are quite damaged by the harsh treatment with detergent and proteases.

In these experiments we used the double promoter plasmid pCMSGFP-VDAC1HaDEVDHis, therefore the distribution of the GFP fluorescence was followed to ensure that plasma membrane permeabilization took place. When the GFP signal was lost in >95% of cells, they were fixed and immunostained to reveal the His and HA tags in hVDAC1-HaDEVDHis (Figure 4). Confocal analysis of the double HA/His staining revealed that, in most of the cells, both tags are still detectable even next to proteinase K digestion (Figure 4C) or trypsin digestion (not shown), suggesting again that the C-terminus of VDAC is exposed toward the IMM space. Anti-cytochrome c immunofluorescence was used, in the same experiment, to confirm that MOM was kept intact, since, in the conditions here used, the intermembrane space was not accessible to proteases (Figure 4D). 0.05% Triton X-100 was instead used to disrupt the MOM, as a positive control. In the presence of Triton X-100 the incubation with proteases produced the loss of the HA and His tags. In the same experiment the endogenous cytochrome c became undetectable by immunofluorescence, meaning that proteases have reached the intermembrane space (Figure 4E represents the HA and cytochrome c immunostaining)(see also Figure S1A).

**Figure 5:**
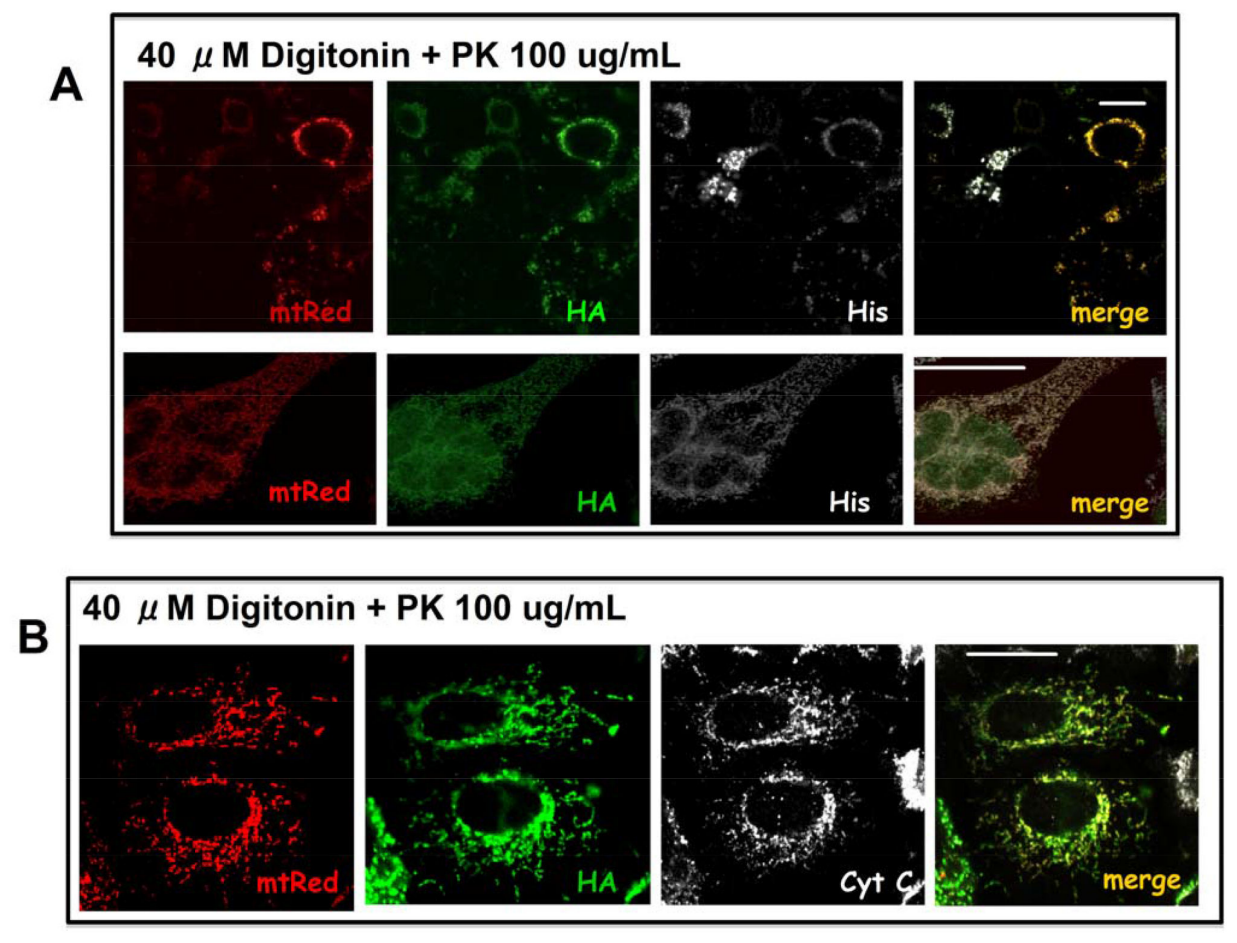
FPP assay upon transfection with pCMSmtDsRed-VDAC1HaDEVDHis. **A**) HeLa cells transfected with pCMSmtDsRed-VDAC1HaDEVDHis. Transfected cells were identified through the mitochondrial fluorescent signal of mtDsRed (red). The incubation with of 40 µM digitonin plus proteinase K does not affect the HA (green) and His (white) staining. **B**) The same as in A, but cytochrome c was stained instead of the His tag, showing its co-localization with VDAC (HA tag in green). The stable localization of cytochrome c demonstrates that the intermembrane space is not accessible to proteases in conditions here used. Images are representative of 50 cells analyzed for each condition in experiments performed in triplicate. Scale bar, 20 µm.

The same experiment was also repeated by using the double promoter plasmid pCMSmtRed-VDAC1HaDEVDHis, which also allows to follows the status of mitochondria (Figure 5A). Immunofluorescence against the endogenous cytochrome c provides indeed a further control of the intactness of the MOM (Figure 5B).

In conclusion the HA and His tags show the same accessibility than endogenous cytochrome c, used here as a control. This result strongly suggests that the VDAC’s C-terminus, bearing the HA and His tags, is oriented towards the mitochondrial intermembrane space.

**Figure 6:**
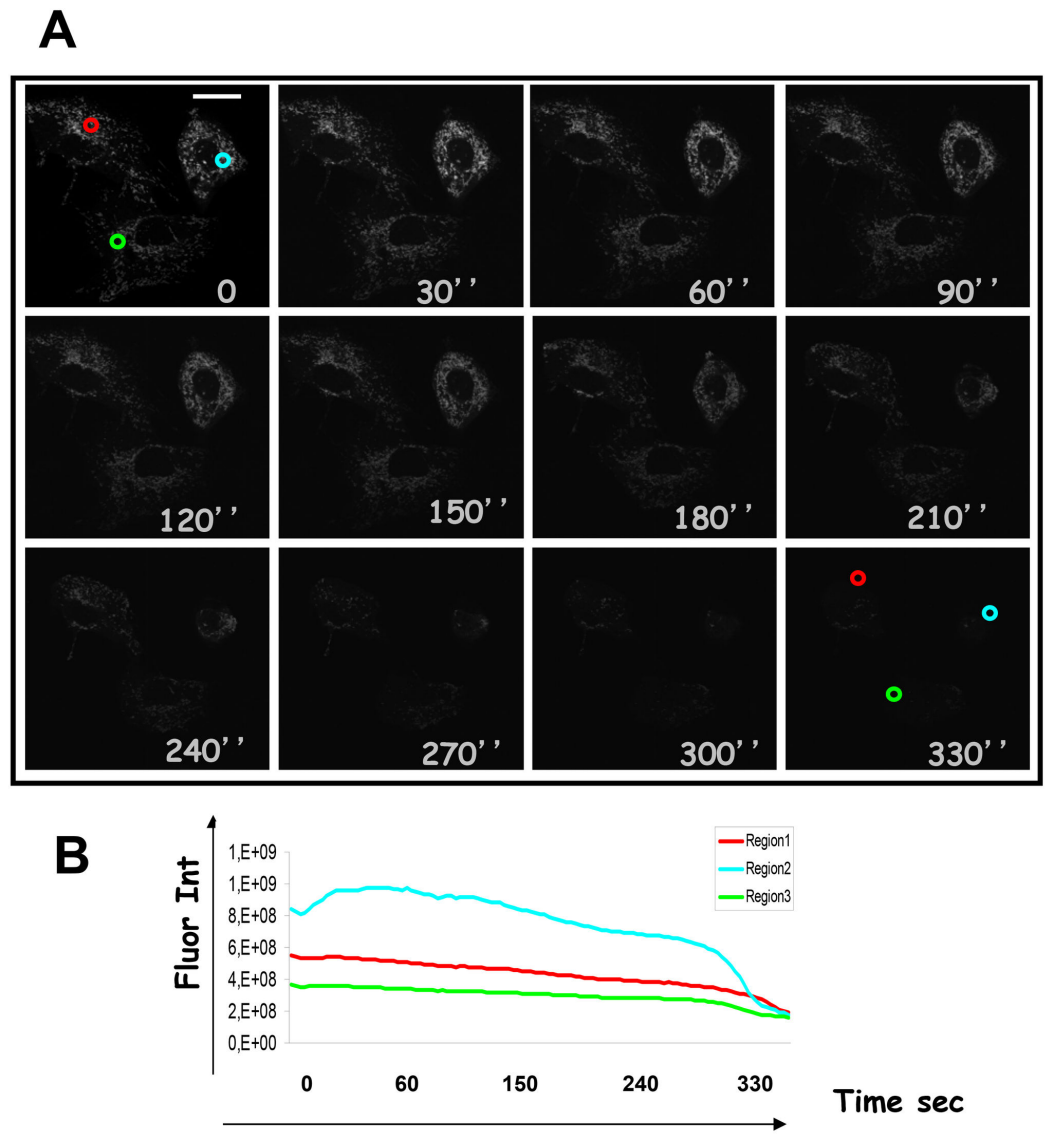
Access of proteases to MomGFP, a transmembrane anchored MOM protein, in the FPP assay. **A**) HeLa cells expressing the mitochondrial outer membrane protein, MomGFP were treated with digitonin and proteinase K as described. Images were taken before and after treatment with digitonin and proteinase K at the indicated time points. The GFP signal becomes weak at 210 seconds and is completely lost at 300 seconds. Scale bar, 20 µm. **B**) Kinetic analysis of the GFP fluorescence fading in three regions of the microscopic field described above.

To validate the assay we have followed, in living HeLa cells, the digestion of the cytosolic moiety of the MomGFP. In MomGFP the GFP anchored to the MOM faces the cytosol so that proteinase K digestion corresponds to the loss of the GFP fluorescence (Figure 6A and B). The experiment clearly shows that GFP fluorescence is lost upon digitonin and proteinase K treatment. In a further control experiment, HeLa cells expressing MomGFP were treated with the digitonin only but without proteinase K. In this case, the GFP signal did not weaken even after 300 seconds. However the permeabilization of the plasma membrane by digitonin, induced the swelling of mitochondria which round up and cluster in the perinuclear region (Figure S3). The FPP assay thus confirms the conclusions obtained by using the caspase cleavage of the DEVD, because when others, general proteases were used, the same result was obtained.

## Discussion

In this work we have demonstrated the topography of VDAC in the outer mitochondrial membrane, showing that the C-terminus faces the intermembrane space. Previous reports have dealt with this topic but an experimental, univocal answer has never been reached before. The novelty of our approach is in the fact that mitochondria were not subject to extraction procedures, potentially harmful for the outer membrane integrity, as in previous works, but the interaction with the probing molecules, i.e. the physiological caspases, was realized inside an intact cell.

Other indications of the C-terminus localization in the intermembrane space are in the literature: this was predicted for Tom40 and Sam50 structures, which are based on the VDAC structure [26]. Moreover recent studies on ubiquitinylation of mitochondrial proteins by Parkin clearly demonstrate the ubiquitinylation of loop residues K53, K274 [27]. This study indirectly indicates the topology of VDAC without the addition of any tag, thus in a physiological condition and thus corroborates our findings. Structural consideration let Bayrhuber et al proposed that the correct orientation of the pore had to resemble the orientation found in all bacterial OM porins [9], and it is as shown here.

**Figure 7:**
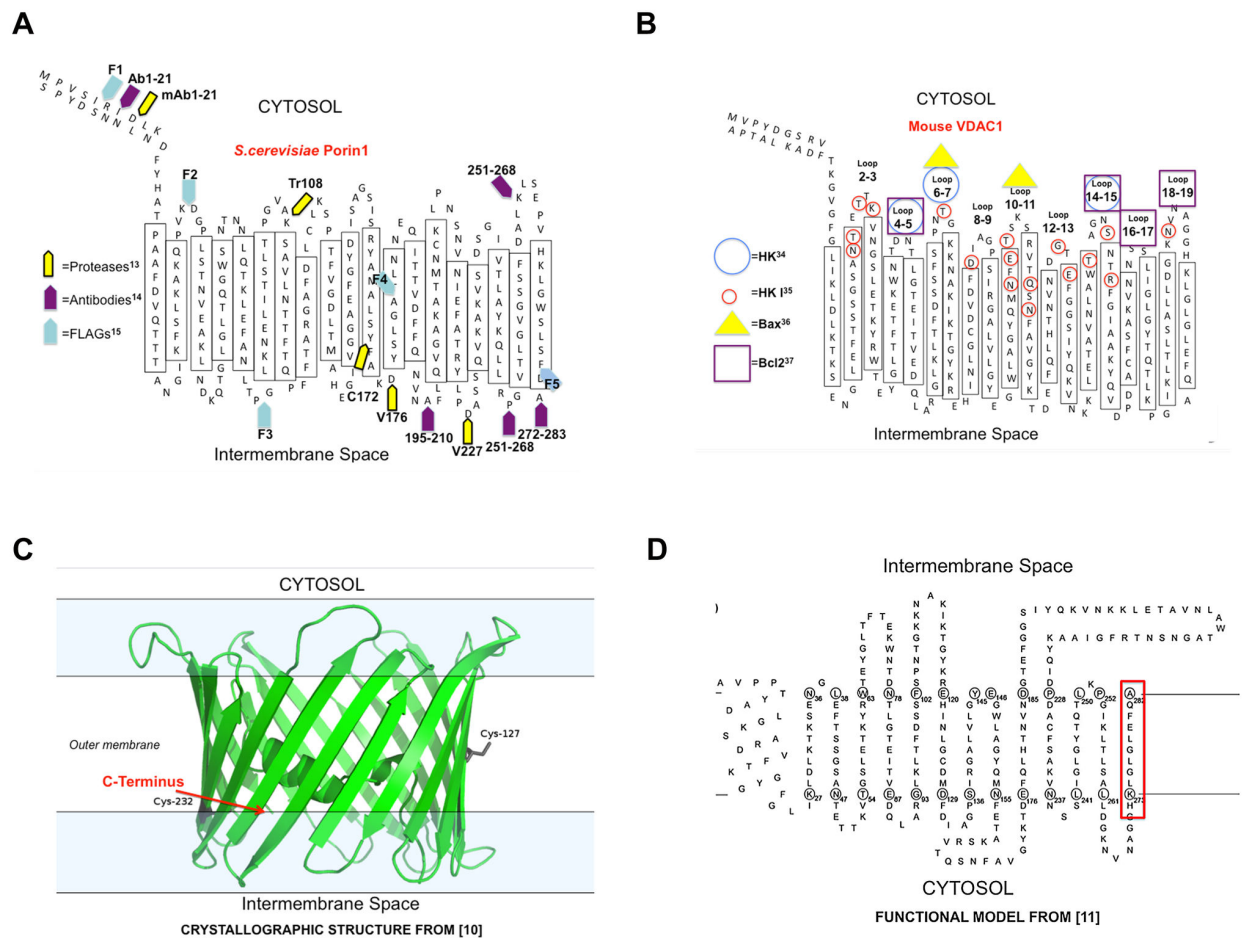
Relevance of VDAC topography: interaction with other proteins. **A**) **Topography of the yeast porin showing the position of proteases and antibodies used for the prediction of sideness**. Yellow arrowheads show the position of the mAb and proteases cleavage sites as described in [13]. Purple arrowheads show the location of epitopes probably detected by the pAb anti-peptides 1-21, 195-210, 251-268, 272-283 [14]. The peptide 251-268 protrudes from both sides of the membrane. This explains its ambiguous results in [14]. The pale blue arrows show the location of FLAG epitopes inserted in yeast porin [15]. **B**) **Topography of the mouse porin showing the position of residues and loops found important in the interaction of VDAC1 with HKI, Bcl-2 and Bax**. Circled in red the residues found involved with the HK-VDAC1 binding [34]; the blue circles mark HKI binding sites as defined by SPR and peptide competition assay [35]; the yellow triangles show the position of peptides anti-Bax [36]; the purple squares mark the loop involved in the binding to Bcl 2 [37]. **C**) **VDAC1 three-dimensional structure**. Lateral view of the protein as reported in pdb: 3emn [10]. The red arrow outlines the C-terminus. Cysteines are indicated. The figure was obtained with PyMol. **D**) **VDAC1** **structure** **as** **defined** **from** **site-directed** **mutagenesis** **experiments** [11]. The red box indicates the C-terminal β-strand.

The topography of VDAC has previously been just a guess, since the number of trans-membrane segments was elusive. Only recently the pore structure has been solved with NMR and crystallography [8-10] stimulating revision of previous information, usually resting on bioinformatic models [28]. Interestingly, the accessibility of exposed loops and turns as it was found in mitochondria by proteases [13] and antibodies [14] is remarkably correct. In the paper by De Pinto et al, the position of cleavage sites for proteases was proposed on the basis of the peptide electrophoretic mobility [13]. The data obtained are consistent with the accessibility of the amino acids to the aqueous phase in the new three-dimensional model, even though the sideness was wrong. In the work by Mannella's group, four antibodies were produced against specific sequences, supposed to be exposed on the basis of bioinformatic models [14]. Two of them reacted with sequences actually found exposed to the intermembrane space, as correctly predicted, while one of them gave uncertain results: this is now explainable, since the peptide against which the Ab was raised spans the membrane [8-10] and has epitopes in both sides of the membrane (Figure 7A). Altogether the work of Mannella's group indicated the correct sideness of the pore, showing that a good biochemistry is a long-lasting value. McDonald and co-workers have recently proposed the topography of VDAC, based upon the three-dimensional models. FLAG-tags were inserted into *S. cerevisiae* VDAC (scVDAC1) and their exposure in intact and disrupted mitochondria detected by immunoprecipitation [15]. The access to such epitopes by antibodies was used as a criterion to propose the pore sideness. Their results could not unambiguously reach a definite conclusion, probably due to the problems in getting intact mitochondria. In particular they suggested that scVDAC1 has its C-terminus exposed to the cytoplasm. In Figure 7A the localization of the FLAGs is reported: FLAGs 2, 3 and 5 location does not agree with our results. It remains only one still puzzling data: the accessibility of the N-terminus to antibodies. This is a very solid result in the literature [14-16,29-32] but it cannot easily be explained with the three-dimensional structure, unless a noteworthy mobility of the N-terminus is supposed. Such mobility is supported by the NMR structure [8], obtained at a physiological temperature, while crystal models were obtained at cryogenic temperatures [9,10]. This is also the reason that convinced us to tag the C-terminus and not the N-terminus. In any case this question does not affect the result and the topography of the pore, as demonstrated in this paper.

The topography of VDAC is highly relevant with respect to its role as a "docking-site" for other proteins. The main examples are Hexokinase and the proteins of the Bcl-2 family. Hexokinase-binding protects against apoptosis but, on the opposite, favours cancer metabolism (Warburg effect) [33]. The binding of HK to VDAC1 has been mapped by surface plasmon resonance (SPR) and competition assay with peptides mimicking various sequences of the pore. The loops 4-5 and, to a lesser extent, 6-7 and 14-15 were found effective in the inhibition of the interaction [34]. These loops indeed are both considered exposed to the cytoplasmic side as defined in this work (Figure 7B). A bioinformatic docking analysis of the HKI-VDAC1 contact has defined a network of atoms candidate to be responsible of the interaction [35]: once more the residues found in this computational analysis are located on the cytosolic side as defined here. Recently a mass-spectrometry analysis has shown that VDAC isoforms on the surface of mitochondria specifically recruit Parkin, showing again the importance of these proteins as harbours for cytoplasmic effectors [27].

Accumulated evidence indicates that both anti- and pro-apoptotic proteins interact with the mitochondrial VDAC, to regulate mitochondria-mediated apoptosis. Two antibodies against peptides (shown in this work to correspond to loops 6-7 and 10-11 (Figure 7B)) were found to prevent Bax- induced loss of the mitochondrial potential [36]. These results suggested that anti-VDAC antibodies block Bax association with mitochondria, Bax-VDAC interaction and permit to define the region of interaction between VDAC and Bax [36]. The most accurate map of the interactions between VDAC1 and Bcl-2 has been obtained by Bcl-2/VDAC1 competitive assay with synthetic peptides. Such peptides were found to completely prevent Bcl-2 mediated protection against staurosporine induced apoptosis [37]. They also interacted directly with Bcl-2 as revealed by SPR technology [37].

Altogether these data confirm the topological sideness of VDAC1 proposed in this work (Figure 7C-D) (see also 38). We offer here a working model to study the interactions of the OM pore forming VDAC1 with cytoplasmic proteins, an urgent task in basic and cancer-drug oriented research.

## Supporting Information

Figure S1 **pCMSmtDsRed-VDAC1HaDEVDHis colocalizes with mitochondrial proteins.** **A**) HeLa cells were transfected with pCMSmtDsRed-VDAC1HaDEVDHis and double-immunostained for the HA tag and the endogenous cytochrome c. pCMSmtDsRed-VDAC1HaDEVDHis transfection results in the simultaneous expression of both mitochondrial hVDAC1-HaDEVDHis (HA, green) and the mitochondrial mtDsRed (red) used as transfection and targeting reporter. Fluorescence distribution analysis indicates the co-localization of VDAC with both the mtDsRed and the cytochrome c in the mitochondrion. **B**) The same as in A. The dot plot, in the last panel on the right, depicts the fluorescence correlation between the red and green signals. Images are representative of 50 cells analyzed for each condition in experiments performed in triplicate. Scale bar, 20 µm.
(TIF)

Figure S2 **Mitochondria are functional in early staurosporine-induced apoptosis.** **A**) Co-transfection of HeLa cells with both pcDNA3-VDAC1HaDEVDHis and MomDEVDGFP. Images were obtained after fixation and immunostaining for the His tag (white). Red stains for MitoTracker (mitochondria), here used as a ΔΨ reporter. We did not measure loss of membrane potential in VDAC transfected cells. **B**) The same as in A, but apoptosis was induced by mild staurosporine treatment as confirmed by the diffusion of the GFP signal in MomDEVDGFP. In the upper panel the cytochrome c is revealed instead of the His tag. In this condition the ΔΨ was maintained even when the cytochrome c was released (upper panel) and the His tag is still detectable (lower panel). **C**) The same as in A, but apoptosis induced by 1 µM staurosporine treatment caused the diffusion of the GFP signal (MomDEVDGFP). Some cells have lost ΔΨ and does not stain for the His tag.
(TIF)

Figure S3 **Control experiment to test the access of proteases to MomGFP in the FPP assay.** **A**) In order to obtain a control for the experiment described in Figure 6, HeLa cells expressing the mitochondrial outer membrane protein, MomGFP were treated with digitonin alone (without proteinase K). Images were taken before (0) and after treatment with 40 μM digitonin at the indicated time points. The GFP signal does not weaken following the digitonin load, even after 300 seconds. However the permeabilization of the plasma membrane by digitonin, induced the swelling of mitochondria which round up and cluster in the perinuclear region. Scale bar, 20 µm. **B**) Kinetic analysis of the GFP fluorescence in three regions of the microscopic field described above.
(TIF)

## References

[1] Shoshan-Barmatz V, De Pinto V, Zweckstetter M, Raviv Z, Keinan N et al. (2010) VDAC, a multi- functional mitochondrial protein regulating cell life and death. Mol Aspects Med 31: 227-285. doi:10.1016/j.mam.2010.03.002. PubMed: 20346371. 20346371

[2] Messina A, Reina S, Guarino F, De Pinto V (2012) VDAC isoforms in mammals. Biochim Biophys Acta 181: 1466-1476. PubMed: 22020053. 10.1016/j.bbamem.2011.10.005 22020053

[3] Rostovtseva T, Colombini M (1997) VDAC channels mediate and gate the flow of ATP: implications for the regulation of mitochondrial function. Biophys J 72: 1954-1962. doi:10.1016/S0006-3495(97)78841-6. PubMed: 9129800. 9129800 PMC1184392

[4] Kroemer G, Galluzzi L, Brenner C (2007) Mitochondrial Membrane Permeabilization in Cell Death. Physiol Rev 87: 99-163. doi:10.1152/physrev.00013.2006. PubMed: 17237344. 17237344

[5] De Stefani D, Bononi A, Romagnoli A, Messina A, De Pinto V, Pinton P, Rizzuto R (2012) VDAC1 selectively transfers apoptotic Ca(2+) signals to mitochondria. Cell Death Differ 19: 267-273. doi:10.1038/cdd.2011.92. PubMed: 21720385. 21720385 PMC3263501

[6] Shoshan-Barmatz V, Mizrachi D (2012) VDAC1: from structure to cancer therapy Front. Oncol 2: 164. 10.3389/fonc.2012.00164 PMC3516065 23233904

[7] Huizing M, Ruitenbeek W, Thinnes F, De Pinto V (1994) Deficiency of the Voltage-Dependent Anion Channel (VDAC): a novel cause of mitochondrial myopathies. Lancet 344: 762. doi:10.1016/S0140-6736(94)92258-6. 7521503

[8] Hiller S, Garces RG, Malia TJ, Orekhov VY, Colombini M, Wagner G (2008) Solution structure of the integral human membrane protein VDAC-1 in detergent micelles. Science 321: 1206-1210. doi:10.1126/science.1161302. PubMed: 18755977. 18755977 PMC2579273

[9] Bayrhuber M, Meins T, Habeck M, Becker S, Giller K et al. (2008) Structure of the human voltage- dependent anion channel. Proc Natl Acad Sci U S A 105: 15370-15375. doi:10.1073/pnas.0808115105. PubMed: 18832158. 18832158 PMC2557026

[10] Ujwal R, Cascio D, Colletier JP, Faham S, Zhang J et al. (2008) The crystal structure of mouse VDAC1 at 2.3 angstrom resolution reveals mechanistic insights into metabolite gating. Proc Natl Acad Sci U S A 105: 17742-17747. doi:10.1073/pnas.0809634105. PubMed: 18988731. 18988731 PMC2584669

[11] Colombini M (2009) The published 3D structure of the VDAC channel: native or not? Trends Biochem Sci 34: 382-389. doi:10.1016/j.tibs.2009.05.001. PubMed: 19647437. 19647437

[12] Hiller S, Abramson J, Mannella C, Wagner G, Zeth K (2010) The 3D structures of VDAC represent a native conformation. Trends Biochem Sci 35: 514-521. doi:10.1016/j.tibs.2010.03.005. PubMed: 20708406. 20708406 PMC2933295

[13] De Pinto V, Prezioso G, Thinnes F, Link TA, Palmieri F (1991) Peptide-specific antibodies and proteases as probes of the transmembrane topology of the bovine heart mitochondrial porin. Biochemistry 30: 10191-10200. doi:10.1021/bi00106a017. PubMed: 1718414. 1718414

[14] Stanley S, Dias JA, D’Arcangelis D, Mannella CA (1995) Peptide-specific antibodies as probes of the topography of the voltage-gated channel in the mitochondrial outer membrane of Neurospora crassa. J Biol Chem 270: 16694-16700. doi:10.1074/jbc.270.28.16694. PubMed: 7542652. 7542652

[15] McDonald BM, Wydro MM, Lightowlers RN, Lakey JH (2009) Probing the orientation of yeast VDAC1 in vivo. FEBS Lett 583: 739-742. doi:10.1016/j.febslet.2009.01.039. PubMed: 19185576. 19185576

[16] Pastorino JG, Hoek JB (2008) Regulation of hexokinase binding to VDAC. J Bioenerg Biomembr 40: 171-178. doi:10.1007/s10863-008-9148-8. PubMed: 18683036. 18683036 PMC2662512

[17] Geula S, Ben-Hail D, Shoshan-Barmatz V (2012) Structure-based analysis of VDAC1: N- terminus location, translocation, channel gating and association with anti-apoptotic proteins. Biochem J 444: 475-485. doi:10.1042/BJ20112079. PubMed: 22397371. 22397371

[18] Schembri L, Zanese M, Depierre-Plinet G, Petit M, Elkaoukabi-Chaibi A, Tauzin L et al. (2009) Recombinant differential anchorage probes that tower over the spatial dimension of intracellular signals for high content screening and analysis. Anal Chem 81: 9590-9598. doi:10.1021/ac9015227. PubMed: 19873978. 19873978

[19] Borgese N, Gazzoni I, Barberi M, Colombo S, Pedrazzini E (2001) Targeting of a tail-anchored protein to endoplasmic reticulum and mitochondrial outer membrane by independent but competing pathways. Mol Biol Cell 12: 2482-2496. doi:10.1091/mbc.12.8.2482. PubMed: 11514630. 11514630 PMC58608

[20] Lorenz H, Hailey DW, Lippincott-Schwartz J (2006) Fluorescence protease protection of GFP chimeras to reveal protein topology and subcellular localization. Nat Methods 3: 205-210. doi:10.1038/nmeth857. PubMed: 16489338. 16489338

[21] Tomasello F, Messina A, Lartigue L, Schembri L, Medina C, Reina S et al. (2009) Outer membrane VDAC1 controls permeability transition of the inner mitochondrial membrane in cellulo durings stress-induced apoptosis. Cell Res 19: 1363-1376. doi:10.1038/cr.2009.98. PubMed: 19668262. 19668262

[22] Ricci JE, Gottlieb RA, Green DR (2003) Caspase-mediated loss of mitochondrial function and generation of reactive oxygen species during apoptosis. J Cell Biol 160: 65-75. doi:10.1083/jcb.200208089. PubMed: 12515825. 12515825 PMC2172744

[23] Schembri L, Dalibart R, Tomasello F, Legembre P, Ichas F et al. (2007) The HA tag is cleaved and loses immunoreactivity during apoptosis. Nat Methods. 4: 107-108. doi:10.1038/nmeth0207-107. PubMed: 17264856. 17264856

[24] Liscum L, Munn NJ (1999) Intracellular cholesterol transport. Biochim Biophys Acta 1438: 19-37. doi:10.1016/S1388-1981(99)00043-8. PubMed: 10216277. 10216277

[25] Plutner H, Davidson HW, Saraste J, Balch WE (1992) Morphological analysis of protein transport from the ER to Golgi membranes in Digitonin-permeabilized cells: role of the P58 containing compartment. J Cell Biol 119: 1097-1116. doi:10.1083/jcb.119.5.1097. PubMed: 1447290. 1447290 PMC2289727

[26] Zeth K (2010) Structure and evolution of mitochondrial outer membrane proteins of beta-barrel topology. Biochim Biophys Acta 1797: 1292-1299. doi:10.1016/j.bbabio.2010.04.019. PubMed: 20450883. 20450883

[27] Sun Y, Vashisht AA, Tchieu J, Wohlschlegel JA, Dreier L (2012) Voltage-dependent anion channels (VDACs) recruit Parkin to defective mitochondria to promote mitochondrial autophagy. J Biol Chem 287: 40652-40660. doi:10.1074/jbc.M112.419721. PubMed: 23060438. 23060438 PMC3504778

[28] De Pinto V, Reina S, Guarino F, Messina A (2008) The structure of Voltage-Dependent Anion selective Channel: state of the art. J Bioenerg Biomembr 40: 139-147. doi:10.1007/s10863-008-9140-3. PubMed: 18668358. 18668358

[29] Babel D, Walter G, Götz H, Thinnes FP, Jürgens L et al. (1991) Studies on human porin. VI. Production and characterization of eight monoclonal mouse antibodies against the human VDAC "Porin 31HL" and their application for histotopological studies in human skeletal muscle. Biol Chem Hoppe Seyler 372: 1027-1034. doi:10.1515/bchm3.1991.372.2.1027. PubMed: 1724155. 1724155

[30] Konstantinova SA, Mannella CA (1995) Immunoelectron microscopic study of the distribution of porin on outer membranes of rat heart mitochondria. J Bioenerg Biomembr 27: 93-99. doi:10.1007/BF02110336. PubMed: 7543088. 7543088

[31] Mannella CA (1998) Conformational changes in the mitochondrial channel protein, VDAC, and their functional implications. J Struct Biol 121: 207-218. doi:10.1006/jsbi.1997.3954. PubMed: 9615439. 9615439

[32] Reina S, Palermo V, Guarnera A, Guarino F, Messina A, et al. (2010) Swapping of the N-terminus of VDAC1 with VDAC3 restores full activity of the channel and confers anti-aging features to the cell. FEBS Lett 584: 2837-2844. doi:10.1016/j.febslet.2010.04.066. PubMed: 20434446. 20434446

[33] Pedersen PL (2007) Warburg, me and Hexokinase 2. J Bioenerg Biomembr 39: 211-222. doi:10.1007/s10863-007-9094-x. PubMed: 17879147. 17879147

[34] Arzoine L, Zilberberg N, Ben-Romano R, Shoshan-Barmatz V (2009) Voltage-dependent anion channel 1-based peptides interact with hexokinase to prevent its anti-apoptotic activity. J Biol Chem 284: 3946-3955. PubMed: 19049977. 19049977 10.1074/jbc.M803614200

[35] Rosano C (2011) Molecular model of hexokinase binding to the outer mitochondrial membrane porin (VDAC1): Implication for the design of new cancer therapies. Mitochondrion 11: 513-519. doi:10.1016/j.mito.2011.01.012. PubMed: 21315184. 21315184

[36] Shimizu S, Matsuoka Y, Shinohara Y, Yoneda, Tsujimoto Y (2001) Essential role of voltage- dependent anion channel in various forms of apoptosis in mammalian cells. J Cell Biol 152: 237-250. doi:10.1083/jcb.152.2.237. PubMed: 11266442. 11266442 PMC2199613

[37] Arbel N, Shoshan-Barmatz V (2010) Voltage-dependent anion channel 1-based peptides interact with Bcl-2 to prevent antiapoptotic activity. J Biol Chem 285: 6053-6062. doi:10.1074/jbc.M109.082990. PubMed: 20037155. 20037155 PMC2825399

[38] Reina S, Magrì A, Lolicato M, Guarino F, Impellizzeri A, Maier E, Benz R, Ceccarelli M, De Pinto V, Messina A (2013) Deletion of β-strands 9 and 10 converts VDAC1 voltage-dependence in an asymmetrical process. Biochim Biophys Acta 1827: 793-805. doi:10.1016/j.bbabio.2013.03.007. PubMed: 23541892. 23541892

