# Supplementary figures and images for: The Voltage-Dependent Anion Selective Channel 1 (VDAC1) Topography in the Mitochondrial Outer Membrane as Detected in Intact Cell
Source: PLoS One. 2013 Dec 6;8(12):e81522. doi: 10.1371/journal.pone.0081522 (PMC3855671; doi:10.1371/journal.pone.0081522)

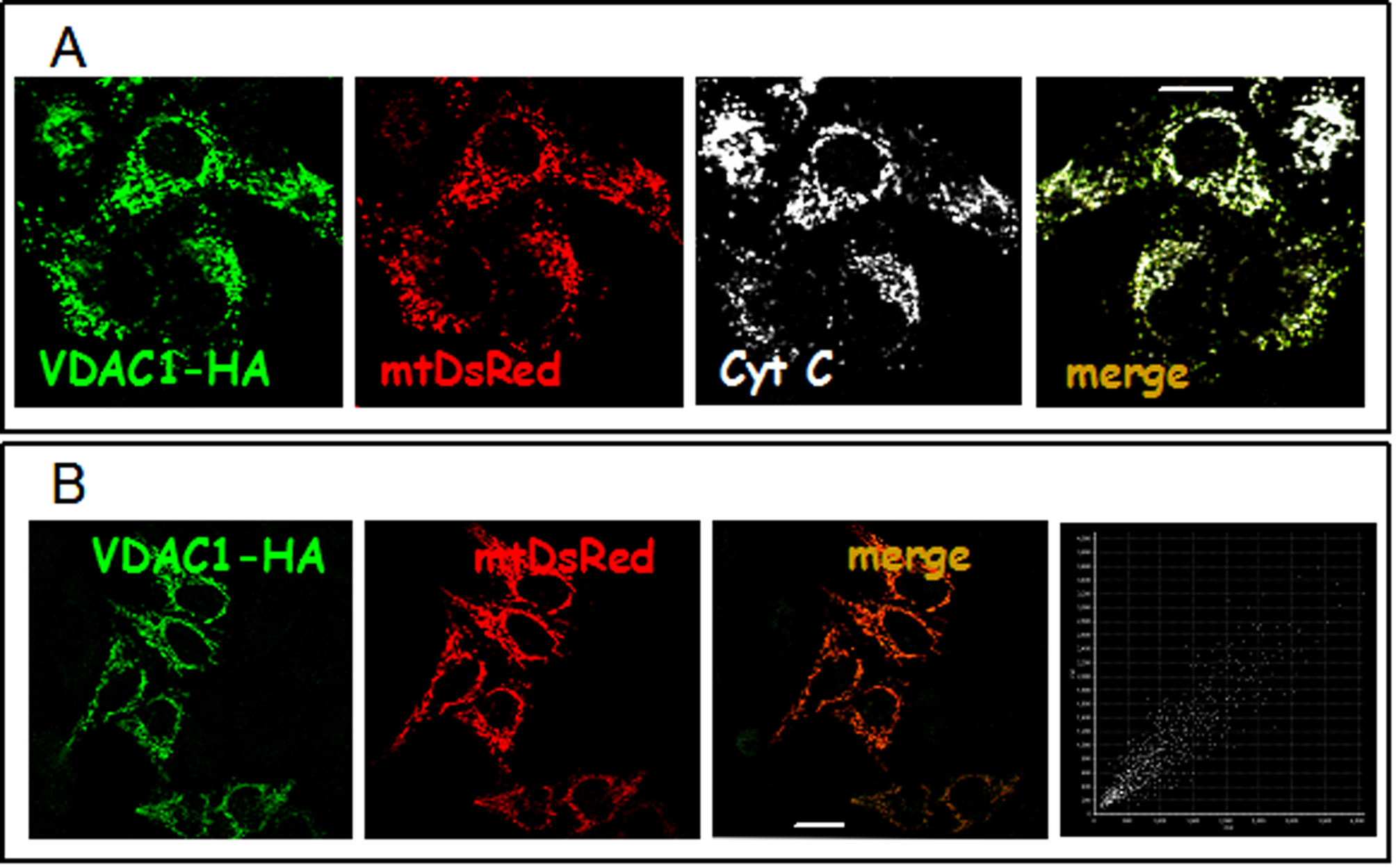

Supplement: Figure S1 — pCMSmtDsRed-VDAC1HaDEVDHis colocalizes with mitochondrial proteins. A) HeLa cells were transfected with pCMSmtDsRed-VDAC1HaDEVDHis and double-immunostained for the HA tag and the endogenous cytochrome c. pCMSmtDsRed-VDAC1HaDEVDHis transfection results in the simultaneous expression of both mitochondrial hVDAC1-HaDEVDHis (HA, green) and the mitochondrial mtDsRed (red) used as transfection and targeting reporter. Fluorescence distribution analysis indicates the co-localization of VDAC with both the mtDsRed and the cytochrome c in the mitochondrion. B) The same as in A. The dot plot, in the last panel on the right, depicts the fluorescence correlation between the red and green signals. Images are representative of 50 cells analyzed for each condition in experiments performed in triplicate. Scale bar, 20 µm. (TIF) [file pone.0081522.s001.tif]

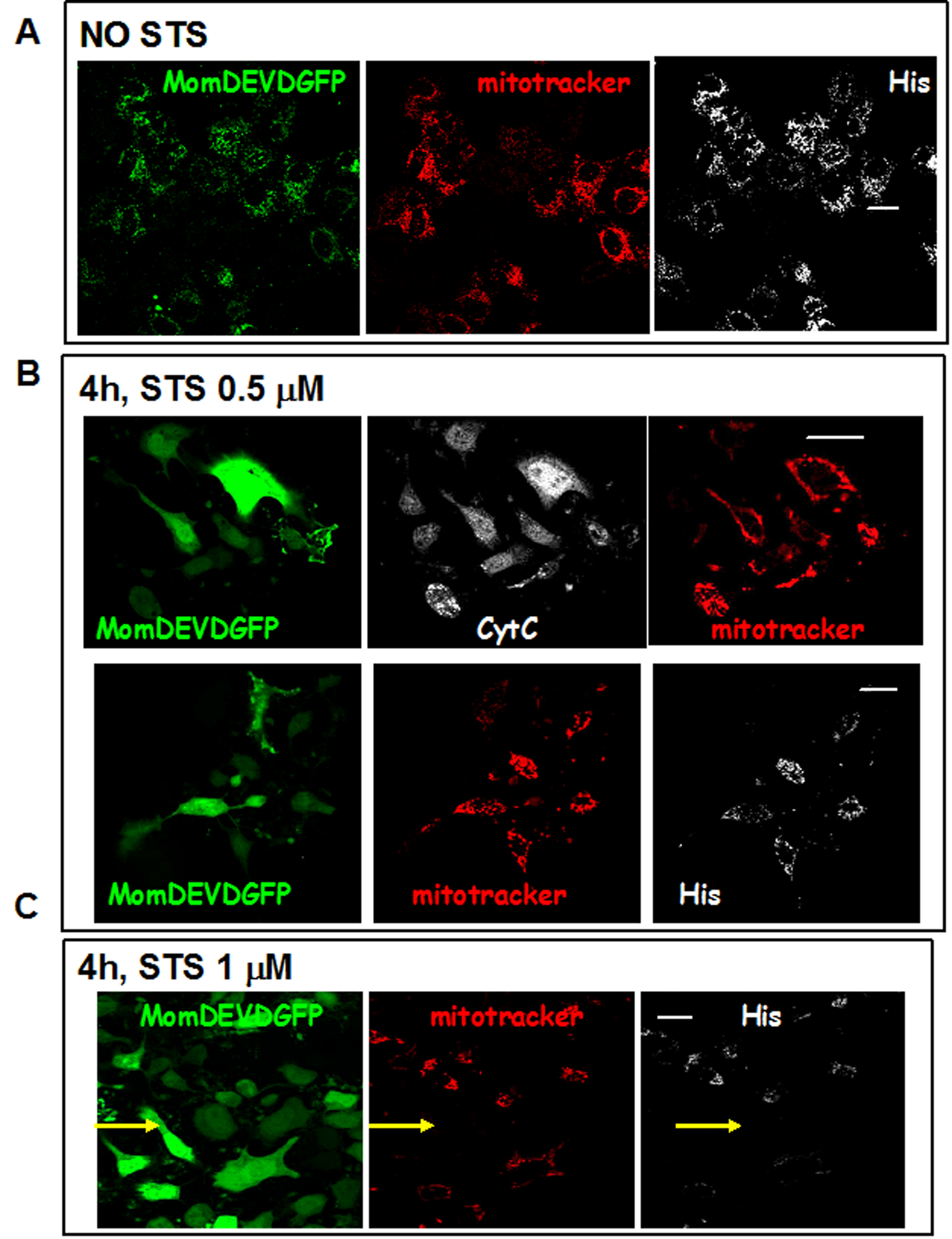

Supplement: Figure S2 — Mitochondria are functional in early staurosporine-induced apoptosis. A) Co-transfection of HeLa cells with both pcDNA3-VDAC1HaDEVDHis and MomDEVDGFP. Images were obtained after fixation and immunostaining for the His tag (white). Red stains for MitoTracker (mitochondria), here used as a ΔΨ reporter. We did not measure loss of membrane potential in VDAC transfected cells. B) The same as in A, but apoptosis was induced by mild staurosporine treatment as confirmed by the diffusion of the GFP signal in MomDEVDGFP. In the upper panel the cytochrome c is revealed instead of the His tag. In this condition the ΔΨ was maintained even when the cytochrome c was released (upper panel) and the His tag is still detectable (lower panel). C) The same as in A, but apoptosis induced by 1 µM staurosporine treatment caused the diffusion of the GFP signal (MomDEVDGFP). Some cells have lost ΔΨ and does not stain for the His tag. (TIF) [file pone.0081522.s002.tif]

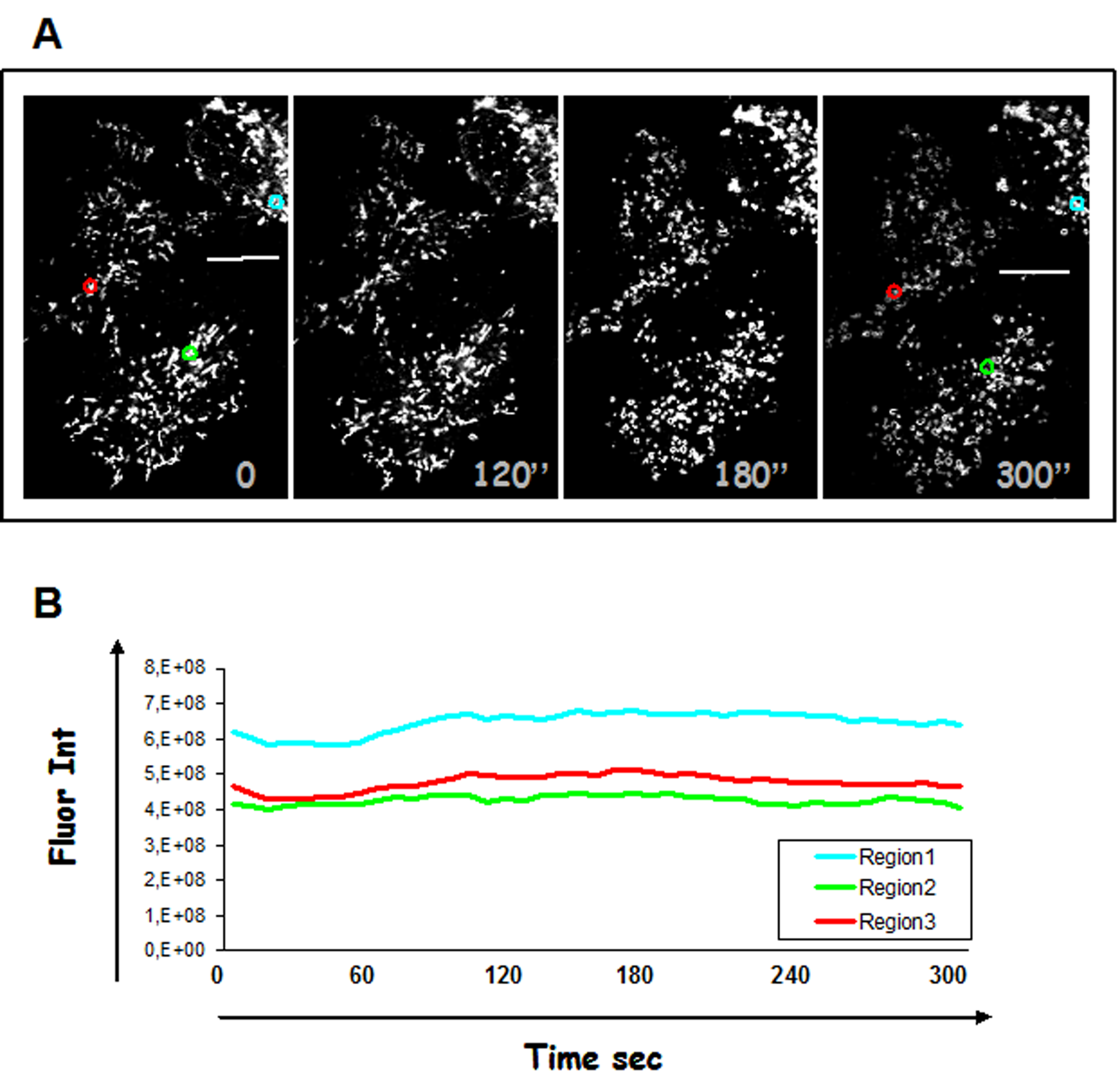

Supplement: Figure S3 — Control experiment to test the access of proteases to MomGFP in the FPP assay. A) In order to obtain a control for the experiment described in Figure 6, HeLa cells expressing the mitochondrial outer membrane protein, MomGFP were treated with digitonin alone (without proteinase K). Images were taken before (0) and after treatment with 40 μM digitonin at the indicated time points. The GFP signal does not weaken following the digitonin load, even after 300 seconds. However the permeabilization of the plasma membrane by digitonin, induced the swelling of mitochondria which round up and cluster in the perinuclear region. Scale bar, 20 µm. B) Kinetic analysis of the GFP fluorescence in three regions of the microscopic field described above. (TIF) [file pone.0081522.s003.tif]
